# Mortality From Parkinson’s Disease With Influenza and Pneumonia as Contributing Causes in the United States, 1999-2020

**DOI:** 10.7759/cureus.92991

**Published:** 2025-09-23

**Authors:** Saima Nazar Salihas, Karthick Muthuramalingam, Anushree Ande, Aswathy Suresh, Abhishek Singh Chauhan

**Affiliations:** 1 Internal Medicine, Travancore Medical College, Kerala, IND; 2 General Medicine, Madras Medical College, Chennai, IND; 3 Internal Medicine, Sri Ramachandra Medical College, Chennai, IND; 4 Cardiology, SK Hospital, Thiruvananthapuram, IND; 5 Community Medicine, Amrita School of Medicine, Faridabad, IND

**Keywords:** age-adjusted mortality rate, cdc multiple cause of death (mcd) database influenza, parkinson's disease, pneumonia, retrospective study

## Abstract

Background: Parkinson’s disease (PD) is a progressive neurodegenerative disorder and a leading cause of morbidity and mortality among older adults. Respiratory infections such as influenza and pneumonia further increase mortality due to impaired cough reflex, dysphagia, and reduced immunity. Limited research has examined long-term mortality trends where these infections contribute to PD-related deaths.

Aims and objectives: This study aimed to evaluate the crude and age-adjusted mortality rates (AAMRs) of individuals with PD where influenza or pneumonia were contributing factors.

Methods: We conducted a retrospective study using the CDC Wide-Ranging Online Data for Epidemiologic Research (WONDER) Multiple Cause of Death (MCD) database (1999-2020). Individuals aged ≥45 years with PD (ICD-10: G20) as the underlying cause of death and influenza or pneumonia (ICD-10: J09-J18) as contributing causes were included. Mortality rates were calculated per 1,000,000 population and age-adjusted to the 2000 US Standard Population. Demographic, geographic, and temporal patterns were analyzed using JoinPoint regression.

Results: From 1999 to 2020, 43,644 deaths were identified. Males accounted for 28,831 deaths (66.1%), and White individuals accounted for 40,716 deaths (93.3%). Most deaths occurred in metropolitan areas (35,261 deaths, 80.8%) and nursing homes/long-term care facilities (19,223 deaths, 44%). The crude mortality rate was 16.6 per 1,000,000 population. AAMRs showed a significant overall decline (annual percentage change (APC), -4.41%; p < 0.05). Male mortality remained consistently higher than female mortality (14,813 deaths, 33.9%). Racial disparities were observed, with White individuals showing the greatest burden (40,716 deaths, 93.3%), while Black (1,581 deaths, 3.6%) and Asian or Pacific Islander groups (1,205 deaths, 2.8%) demonstrated fluctuating but declining trends.

Conclusion: Mortality from PD with influenza or pneumonia declined significantly between 1999 and 2020, reflecting improvements in infection control and elderly care. However, disparities by sex, race, and geography persist, underscoring the need for targeted interventions, particularly in long-term care settings.

## Introduction

Parkinson’s disease (PD) is a progressive neurodegenerative disorder affecting about 1% of individuals over 60 years of age and is the second most common neurodegenerative disease after Alzheimer’s disease [1,2]. Characterized by motor symptoms such as bradykinesia, tremor, and rigidity, along with multiple non-motor features, PD significantly impairs independence and quality of life [3].

While PD itself is not directly fatal, it predisposes patients to life-threatening complications. Aspiration pneumonia, driven by dysphagia, impaired swallowing reflex, and reduced cough efficiency, is the leading cause of death among PD patients [4,5]. Mortality studies have shown that individuals with PD are at a three- to four-fold higher risk of dying from pneumonia or influenza compared to the general population [6]. Immobility and impaired airway clearance further amplify this risk [7].

Influenza, responsible for 3-5 million severe cases and nearly 300,000-650,000 respiratory deaths annually worldwide, poses a serious burden among older and immunocompromised populations [8]. Importantly, influenza frequently precipitates pneumonia, and together, these infections are major contributors to PD mortality [4,6]. Beyond acute effects, influenza infection has also been associated with a possible long-term risk of developing PD. A Danish nationwide study (1977-2016) showed that individuals with influenza more than 10 years prior had a significantly higher risk of subsequent PD (OR 1.73, 95% CI 1.11-2.71) [9].

In the United States, influenza and pneumonia together accounted for more than 1.25 million deaths between 1999 and 2020, although overall age-adjusted mortality rates (AAMRs) have declined in this period [10]. However, the specific contribution of influenza and pneumonia to mortality in people with PD remains poorly understood.

Understanding the demographic, geographic, and temporal patterns of PD-related deaths complicated by respiratory infections is vital for targeted public health strategies. This includes vaccination, dysphagia screening, pulmonary rehabilitation, and early antimicrobial interventions in PD populations. Using the CDC Wide-Ranging Online Data for Epidemiologic Research (WONDER) Multiple Cause of Death (MCD) database, this study examines epidemiological trends in individuals aged ≥45 years where PD was the underlying cause of death and influenza or pneumonia were contributing causes between 1999 and 2020 [10].

This study aimed to evaluate the crude and AAMRs in individuals with PD where influenza or pneumonia contributed to death, using the CDC WONDER MCD database from 1999 to 2020. The study stratifies data by gender, race, geographic area, and place of death to identify disparities in mortality patterns.

## Materials and methods

A retrospective original research study was conducted utilizing the CDC WONDER MCD database [10]. Data extraction was performed on June 11, 2025, and as the dataset consists of publicly available, de-identified information, the study was classified as non-human participant research, thus exempt from ethics committee approval [11].

Mortality data were extracted from the CDC WONDER MCD database for the years 1999-2020. The study included individuals aged 25 years and above, as PD-related mortality in younger populations is rare. PD (G20) was selected as the underlying cause of death, while influenza and pneumonia (J09-J18) were selected as the multiple causes of death to assess the co-occurrence of these conditions. Demographic variables such as gender (male and female) and race/ethnicity (American Indian or Alaska Native, Asian or Pacific Islander, Black or African American, or White) were included to analyze disparities in mortality outcomes. Geographic variables included urbanization based on the 2013 classification, categorizing metropolitan areas into large central metro, large fringe metro, medium metro, and small metro, and non-metropolitan areas into micropolitan and non-core rural areas [12]. Additionally, the place of death was categorized as a medical facility, decedent's home, hospice facility, or nursing home/long-term care. Mortality rates were standardized using rates per 1,000,000 population, with adjustments based on the US Standard Population from the year 2000, to allow for accurate comparisons over time [13].

Descriptive statistics, including absolute numbers and percentages, were used to summarize the demographic and geographic variables. AAMRs were generated for each variable using the CDC WONDER MCD and the above criteria. Those records that lacked critical variables as mentioned, such as demography, urbanization, and place of death, were excluded to ensure proper analysis. To evaluate temporal trends, JoinPoint Regression Analysis (JoinPoint Software Version 5.3.0.0, November 2024) was used to determine annual percentage changes (APC) in mortality from 1999 to 2020 in patients with PD with influenza and pneumonia as contributing causes. Trends were assessed over the 1999-2020 study period to identify statistically significant changes in mortality patterns across different demographic and geographic groups.

## Results

From 1999 to 2020, the CDC WONDER Multiple Cause of Death (MCD) database recorded 43,644 deaths in the United States among individuals aged 25 years and older. The study included cases in which PD (ICD-10: G20) was listed as the underlying cause of death and influenza or pneumonia (ICD-10: J09-J18) as multiple causes of death (n = 43,644). The crude mortality rate for PD with influenza and pneumonia as contributing causes was 16.6 per 1,000,000 population. Deaths not meeting these criteria were excluded.

Demographic characteristics

Among the total deaths analyzed, males accounted for 28,831 (66.10%), while females accounted for 14,813 (33.90%). The mortality rate for PD with influenza and pneumonia as contributing causes was higher in males compared to females, indicating a potential demographic disparity. Regarding racial distribution, the highest proportion of deaths occurred among White individuals (40,716, 93.30%), followed by Black or African American individuals (1,581, 3.6%), Asian or Pacific Islander individuals (1,205, 2.8%), and American Indian or Alaska Native individuals (142, 0.3%). The mortality burden was highest among White individuals, highlighting racial disparities in mortality trends related to PD with influenza and pneumonia. Study characteristics are provided in Table 1.

**Table 1:** Demographic characteristics of the study

Demographic variable	Number of deaths (n)	Percentage (%)
Gender		
Male	28,831	66.10
Female	14,813	33.90
Race		
American Indian or Alaska Native	142	0.30
Asian or Pacific Islander	1,205	2.80
Black or African American	1,581	3.60
White	40,716	93.30
Urbanization		
Metropolitan area	35,261	80.8
Large central metro	11,695	26.80
Large fringe metro	10,089	23.10
Medium metro	9,210	21.10
Small metro	4,267	9.80
Non-metropolitan area	8,383	19.2
Micropolitan	4,829	11.10
Non-core	3,554	8.10
Place of death		
Medical facility	15,997	36.65
Decedent's home	5,211	11.94
Hospice facility	1,767	4.05
Nursing home/long-term care	19,223	44.05
Other	1,446	3.31

Geographic characteristics

The majority of deaths occurred in metropolitan areas (35,261, 80.8%), while non-metropolitan areas accounted for 8,383 (19.2%) deaths. Regarding the place of death, most deaths occurred in medical facilities (15,997, 36.65%), followed by decedents' homes (5,211, 11.94%), nursing homes or long-term care facilities (19,223, 44.05%), and hospice facilities (1,767, 4.05%).

Temporal trends

**Figure 1:**
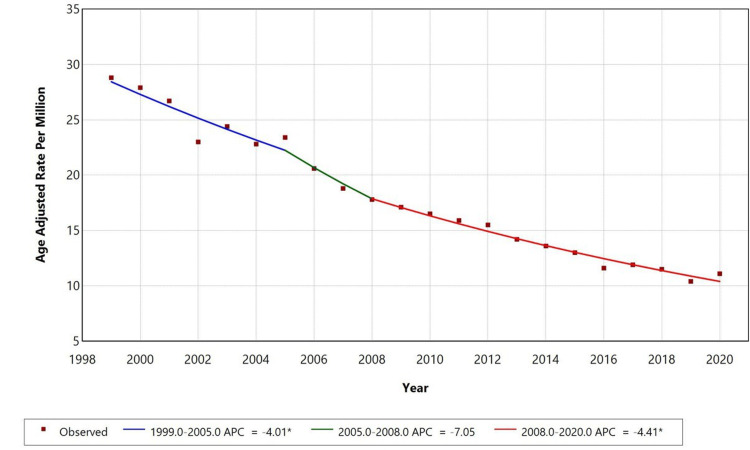
Overall age-adjusted mortality rates among adults aged ≥45 in the United States, 1999-2020 *Annual percentage change (APC) is statistically significant at α = 0.05.

From 1999 to 2020, the AAMR for PD with influenza and pneumonia as contributing causes showed a declining trend. The APC declined from 1999 to 2005 (-4.01, p < 0.05) and again from 2008 to 2020 (-4.41, p < 0.05). The interval between 2005 and 2008 did not show a statistically significant change, which is why the JoinPoint model did not assign a separate APC segment for that period. This has been clarified to ensure consistent reporting of statistical significance across time segments and demographic groups (Figure 1).

**Figure 2:**
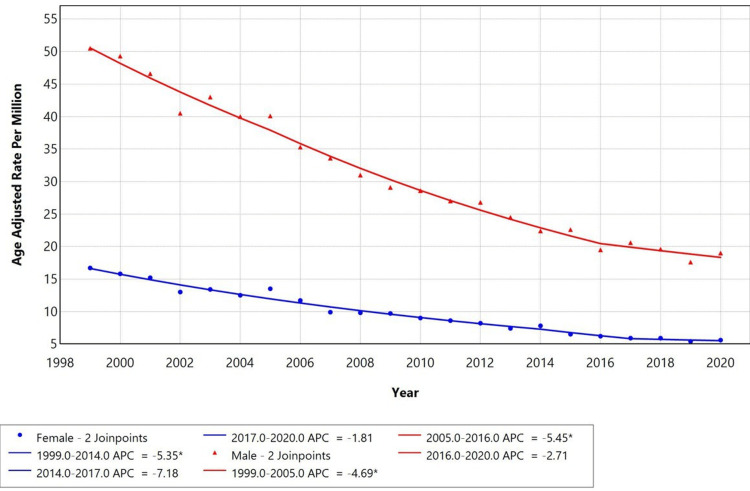
Trends in sex-stratified age-adjusted mortality rates among adults aged ≥45 in the United States, 1999-2020 *Annual percentage change (APC) is statistically significant at α = 0.05.

When stratified by gender, males had a higher AAMR with an APC of -4.69 from 1999 to 2005, with a further significant decline from 2005 to 2016 with an APC of -5.45, compared to females' AAMR with an APC of -5.35 from 1999 to 2014. However, both groups exhibited a similar declining trend with APCs of -5.45 (p < 0.05) for males and -5.35 for females (p < 0.05), respectively, as shown in Figure 2.

**Figure 3:**
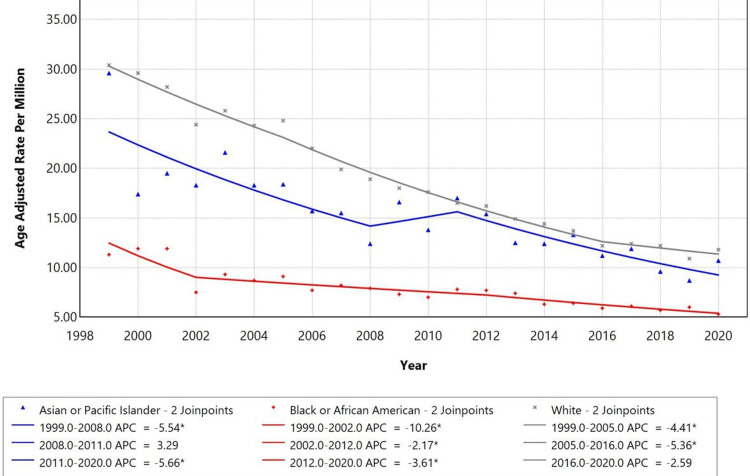
Trends in race-stratified age-adjusted mortality rates among adults aged ≥45 years in the United States, 1999-2020 *Annual percentage change (APC) is statistically significant at α = 0.05.

Racial disparities were observed in PD with influenza and pneumonia as contributing causes for mortality trends. White race had the highest AAMR with an APC of -4.41 from 1999 to 2005, with a further decline to -5.36 from 2005 to 2016, followed by Asians or Pacific Islander population, showing an APC of -5.54 from 1999 to 2008 and an APC of -5.66 from 2011 to 2020, and Black or African American group, showing steep declining patterns with an APC of -10.26 in the earlier period (1999-2002). The trends remained declining further from 2002 to 2012, with an APC of -2.17 and -3.61 (p < 0.05). The temporal trends for the American Indian/Alaska Native Islander group are not displayed due to data suppression for counts <10, limiting reliable trend analysis, as represented in Figure 3.

## Discussion

This retrospective study was conducted using data from the CDC WONDER MCD database to assess mortality trends among individuals aged 45 years and older with PD (ICD-10: G20) as the underlying cause of death and influenza or pneumonia (ICD-10: J09-J18) as contributing causes in the United States between 1999 and 2020. A total of 43,644 deaths were identified over the 22-year period. The AAMR showed a gradual overall decline, falling from 4.49 per million in 1999 to 3.22 per million in 2020, despite some fluctuations in the intervening years. Males exhibited higher mortality than females, and White individuals accounted for 93.30% of deaths. Geographic analysis revealed that more than 80% of deaths occurred in metropolitan areas, particularly large central metros, while non-metropolitan areas had lower reported rates. These patterns suggest demographic, geographic, and healthcare-related disparities influencing mortality risk.

The interaction between PD and respiratory infections such as influenza or pneumonia is both physiologically and epidemiologically significant. Neurodegeneration in PD impairs swallowing, respiratory muscle function, and cough reflex, increasing the risk of aspiration and respiratory compromise [14]. Infections like influenza or pneumonia can further exacerbate this vulnerability, triggering systemic inflammation, hypoxia, and functional decline [15,16]. Epidemiologically, these infections are among the leading causes of hospitalization and mortality in patients with PD, particularly in older adults [15]. The underlying pathogenesis often involves aspiration events, impaired immunity, respiratory failure, and multi-organ dysfunction, contributing substantially to increased morbidity and mortality [14-16].

This study found that PD with influenza or pneumonia as contributing causes of death had an average APC of -4.41% between 1999 and 2020. While national mortality data indicate that overall PD mortality has increased over the past two decades [17], our findings align more closely with research on PD complicated by respiratory infections, which has shown a decline in mortality over similar time frames [18]. This pattern parallels broader declines in influenza and pneumonia mortality in the US population, likely reflecting improvements in respiratory infection prevention, early diagnosis, and acute management [18,19]. However, despite this decline, certain groups continue to face higher mortality risks, emphasizing the need for targeted interventions and continued research into modifiable risk factors. The persistent susceptibility of PD patients to respiratory complications underscores the importance of targeted prevention strategies, including vaccination and dysphagia management, to sustain and further reduce mortality in this high-risk group.

This study revealed marked demographic disparities in mortality among individuals with PD and concurrent respiratory infections. White males had the highest AAMRs, while females consistently exhibited lower rates, likely due to slower disease progression and hormonal protection [20,21]. Racial differences were also notable, with White individuals accounting for 93.30% of total deaths. While this may reflect a higher prevalence of PD in this group, it could also be influenced by differences in diagnostic accuracy, healthcare utilization, and reporting practices [22]. Minority populations may experience underdiagnosis or limited access to specialist care [23]. Broader factors such as genetic predispositions, socioeconomic inequalities, and disparities in long-term care quality likely contribute to the observed trends [23]. These findings emphasize the need for targeted public health interventions and equitable healthcare access to reduce mortality across all demographic groups.

This study identified notable geographic disparities, with over 80% of deaths occurring in metropolitan areas, particularly in large central and fringe metros [24]. Higher mortality in these regions may be associated with the greater concentration of elderly individuals in long-term care facilities, where respiratory infections are frequently reported. However, given the observational design of this study, these findings represent associations rather than causal relationships [25-27]. Improved diagnostic and reporting practices in urban centers may also contribute to higher recorded rates [27]. In contrast, non-metropolitan areas reported fewer deaths, possibly due to underdiagnosis or limited healthcare access [28]. Environmental exposures, lifestyle risk factors, and regional variations in care availability may further influence these patterns, highlighting the need for location-specific health interventions.

Over the 22-year study period, overall AAMRs for PD with respiratory infections declined, reflecting potential improvements in disease management, infection control, and elderly care [18]. However, trends varied across subgroups. Males initially showed a steady decline in mortality, but a slight rise was observed after 2016, whereas females continued to show a consistent downward trend. Racially, White individuals demonstrated a steady decline, while Asian or Pacific Islander and Black populations exhibited fluctuating patterns. These variations may reflect differences in access to care, vaccination uptake, comorbidities, or changes in coding practices over time. Continuous surveillance is essential to understand and address emerging trends.

The observed decline in mortality rates is encouraging but underscores the need to address persistent disparities across gender, race, and geographic regions. Future research should explore treatment accessibility, vaccination uptake, and risk factor modification, particularly in long-term care and underserved populations. Public health strategies should focus on early diagnosis, infection prevention, and equitable care delivery. Tailored interventions at the community and institutional levels are essential to mitigate disparities and improve outcomes for patients with PD.

Limitations

This study has some limitations related to the use of the CDC WONDER MCD database. Death certificate data may be subject to misclassification, coding inaccuracies, and underreporting, which can affect the reliability of cause of death information. The database lacks detail on clinical variables such as disease severity, comorbidities, vaccination history, and treatment regimens. Additionally, it does not include socioeconomic status, healthcare access, or lifestyle factors, which may influence mortality outcomes. Changes in diagnostic criteria and reporting practices over the years may also have impacted trend analysis. As a retrospective observational study, causal inferences cannot be established. Moreover, subgroup analysis, particularly for American Indian/Alaska Native populations, was limited due to data suppression when death counts were fewer than 10, which restricts a full understanding of disparities in smaller populations.

## Conclusions

This retrospective analysis reveals significant declines in AAMRs associated with PD and concurrent influenza or pneumonia from 1999 to 2020 across most demographic groups. Males and White individuals accounted for the highest proportion of deaths, with racial disparities evident in mortality trends. The majority of deaths occurred in metropolitan areas and long-term care facilities, highlighting areas for targeted public health intervention. The consistent downward trend in mortality suggests improved disease management, vaccination efforts, and supportive care, although disparities by sex, race, and geographic location remain. These findings underscore the need for continued surveillance and tailored healthcare strategies to further reduce mortality in this vulnerable population.
